# Biological responses to nanoscale particles

**DOI:** 10.3762/bjnano.6.37

**Published:** 2015-02-05

**Authors:** Reinhard Zellner

**Affiliations:** 1 University of Duisburg-Essen, Institute of Physical Chemistry, Universitätsstraße 5, 45141 Essen, Germany

Nanoscale particles are known to exhibit novel and unprecedented properties that make them quite different from their corresponding bulk-scale materials. The key differences are (i) an increased relative surface area and (ii) quantum size effects. Since the growth, catalytic activity and the various interactions with molecules (including biomolecules) occur at the surface of nanoparticles, a given mass of material in nanoparticle form will be much more reactive than the same mass of bulk material. As the properties of nanoscale materials become better understood and our ability to control these properties is further advanced, an enormous potential to create materials with novel properties and applications emerges. Nanoscience and nanotechnology are set to revolutionize many fields of science.

Although the technological, economic and perhaps even societal benefits of nanomaterials are indisputable, concerns have also been raised that the very same properties that make them interesting might also have negative health effects. Unfortunately, these potentially negative effects cannot easily be predicted or derived from the known toxicity of the corresponding macroscopic material. Hence, major gaps in the knowledge necessary for assessing their risk to human health currently exist. There is also a lack of existing methodologies to improve techniques for nanoparticle characterization, the detection and localization of nanoparticles in biological systems, as well as the biological activity, fate and persistence of such systems. The complexity of this problem is amplified by the huge variety of nanoscale materials and objects as well as the enormous number of potential biomolecules and cells, thus creating a large parameter space to be examined.

With these shortcomings in mind, we initiated a national Priority Program (Schwerpunktprogramm SPP1313) in Germany in 2007 at the Deutsche Forschungsgemeinschaft (DFG) entitled, “Biological Responses to Nanoscale Particles (Bio-Nano-Responses)”. In this research network, a fundamental understanding of interactions between nanoparticles and biological systems at the molecular and cellular level are to be investigated. The major objective has been to elucidate the physical, chemical and biological elementary processes by which manufactured nanoparticles enter a biological environment, interact with its components and interfere with its functions.

In this program, the bio–nano response beginning at an exposure entry port such as the lung, the GI tract or the skin has been studied as a sequence of interactions, namely,

- the interactions with proteins and cellular constituents,
- the transfer across boundaries and biological membranes,
- the intercellular trafficking, and
- the impact on important biological functions and cell constituents.

It was agreed that proper synthesis, thorough purification and full characterization of nanoparticles using state-of-the-art technologies were paramount in order to assess their biological action. Moreover, the aim was to correlate detailed material properties with their biological effects in order to elucidate the biological response to the material challenge. The nanoparticles used in this study were those of current, wide-spread technological importance, such as metals (e.g., silver, gold, platinum), oxides (e.g., silica, iron oxide, cerium oxide, manganese oxide), polymers (e.g., polystyrene) and quantum dots (II/VI semiconductors). Naturally occurring and industrially available nanoparticles have generally not been considered.

The work of this project primarily addressed the behavior of purposely-designed, highly-engineered nanoparticles under conditions of non-intended, accidental exposure to biological environments. Although it is known (from previous toxicological studies of nanoparticles) that the surface area seems to be one of the properties that causes a severe biological response, other properties such as solubility, hydrophobicity, surface functionalization, surface charge, colloidal stability and nanoparticle morphology have been suggested to be of equal relevance. Since it is these properties that are frequently modified in engineered nanoparticles to improve their applicability, the investigation of the interdependence of the bio–nano activity has been of primary importance. Therefore, questions concerning the mechanism of interaction at the molecular and subcellular levels (as well as their consequences for cell integrity and function) constituted the priorities for this research.

In addition to “simple” nanoparticles, some groups in the SPP1313 have also focused on the synthesis of multifunctional particles, in which parameters such as fluorescence, surface conjugates of biomolecules, magnetism, radioactivity, Janus particles and core–shell particles were combined. In particular, the use of fluorescently labeled particles has become one of the preferred tools to track nanoparticles inside cells and tissue.

When nanoparticles are exposed to biological fluids, their surface-active components (i.e., proteins) will adhere to the nanoparticles and form a so-called “protein corona”. As a result, biological systems never come into contact with bare nanoparticles, but rather with their protein-coated analogues. Since this discovery, a large variety of experimental techniques have been developed to unravel the chemical and molecular mechanistic details, as well as their biological consequences.

Depending on whether a given cell spends energy during the uptake of nanoparticles or not, such uptake through the cell membrane is considered to be active or passive. While small molecules or nanoparticles can cross membranes with the mediation of specialized proteins, larger nanoparticles exhibit more complex entry mechanisms that are jointly described as endocytosis.

The entry mechanism of nanoparticles into living cells and their subsequent trafficking within the cell and translocation between cells are important processes which affect not only nanotoxicology but also nano-biomedicine. In the SPP1313, several projects presented experimental strategies to study and dissect nanoparticle uptake. The employed approaches comprised not only technical advancements such as novel imaging techniques and particle tracking algorithms, but also generic molecular ways to interfere with uptake mechanisms by using si-RNAs and chemical inhibitors. However, a solid conclusion on whether or not nanoparticle-specific entry routes exist and/or which of the established routes are preferred as a function of shape, size, and most importantly, surface functionalization and protein corona, could thus far not be established.

The SPP1313 research network resulted from an open national call after an intensive evaluation of proposals by an international group of experts. It operated for a total of 6 years with an intermediate reevaluation after 3 years. An important condition for participation was clustering. The approximately 40 projects in this network were requested to form cooperative and interdisciplinary clusters with 3–4 projects in each cluster, led by a cluster leader. In fact, the successful participation in SPP1313 was not only based on the evaluation of the individual projects but also on the prospective, added value expected from the formation of such clusters.

The idea of clustering was born on the basis of the expectation that “horizontal” cooperation between chemists/material scientists on the one side and biologists/toxicologists on the other side would foster the cooperation and enhance its effectiveness. Hence, clustering largely prevented pure disciplinary work but rather promoted cooperative science. After six years of participation, it was clear that such a structural organization of the network was the key to its success. To date, a total of approximately 250 peer-reviewed papers have been published.

This special issue in the Beilstein Journal of Nanotechnology reflects some of this work. This issue was motivated by the idea that a comprehensive overview over the science conducted in SPP1313 was the most appropriate way to make the results from this network known to a wider group of experts both in material science and the sciences of related biological effects. Because most of the original work from the individual research groups or clusters has already been published, this special issue mostly contains mini-reviews, which summarize the current and previous work of the contributing groups.

The successful operation of a large research network requires the commitment of many people. As a coordinator of the SPP1313 network, I would like to express my sincere gratitude first of all to the Deutsche Forschungsgemeinschaft for funding this network and in particular to Dr. Torsten Hotopp for his favorable and encouraging administration. Additionally, I wish to thank members of the international evaluation board as well as the members of the Scientific Steering Committee of SPP1313, who provided substantial guidance and facilitation in coordinating this network. I would also like to acknowledge the substantial support I have received from the Rector of the University of Duisburg-Essen and the Dean of the Faculty of Chemistry during the course of this program. Finally, it is necessary to mention that without the enormous commitment of my resident colleagues, Dr. Oliver Locker-Grütjen and Dr. Michael Eisinger from ZWU, as well as of my secretaries, Iris Marissen and Natascha Seyock, the coordination of the program would not have been as effective and enjoyable. Lastly, it is my pleasure to acknowledge the excellent cooperation with the Beilstein editorial office for the preparation of this special issue.

Reinhard Zellner

Essen, December 2014

